# Protocol for Mesothelioma Observational study of Risk prediction and Generation of paired benign-meso tissue samples, Including a Nested MRI Substudy (Meso-ORIGINS)

**DOI:** 10.1136/bmjresp-2025-004045

**Published:** 2026-07-01

**Authors:** Mark D J Neilly, Alexandrea MacPherson, Laura Alexander, Nicola Walker, Caroline Kelly, Joshua Roche, Emad Abugassa, Liam Allan, Adeel Ashraf, Avinash Aujayeb, Anna Bibby, Rocco Bilancia, John Corcoran, Mahendran Chetty, Kevin Conroy, Christopher Craig, Rebecca Crook, Alveena D’Souza, Cyrus Daneshvar, Duneesha DeFoneska, Poppy Denniston, Janet Fallon, Katie Ferguson, Timothy Gatheral, Andrew Lloyd Griffiths, Mohammed Haris, Mohammed Hashim, Alina Ionescu, Owais Kadwani, Michelle Macdougall, John D Maclay, Oliver Nesfield Mann, Nick Maskell, Morvern Morrison, Rakesh Panchal, Benjamin Prudon, Najib M Rahman, Rajini Sudhir, Sudarshan Ramsaroop, Raja Reddy, Phil Reid, Elizabeth Sage, Philip Short, Andrew E Stanton, Laura Succony, Georgios Tsaknis, Selina Tsim, Gordon Cowell, Kevin Lamote, Kevin G Blyth

**Affiliations:** 1 Glasgow Pleural Disease Unit, Queen Elizabeth University Hospital, Glasgow, UK; 2 School of Cancer Sciences, University of Glasgow, Glasgow, UK; 3 Glasgow Oncology Clinical Trials Unit, Glasgow, UK; 4 Royal Stoke University Hospital, Stoke-on-Trent, UK; 5 Raigmore Hospital, Inverness, UK; 6 Blackpool Teaching Hospital, Blackpool, UK; 7 Northumbria Specialist Emergency Care Hospital, Northumbria, UK; 8 University of Bristol Medical School, University of Bristol Academic Respiratory Unit, Bristol, UK; 9 Southmead Hospital, Bristol, UK; 10 Golden Jubilee National Hospital, Clydebank, UK; 11 Royal Devon and Exeter Hospital, Exeter, UK; 12 Aberdeen Royal Infirmary, Aberdeen, UK; 13 University Hospital of North Tees, Stockton-on-Tees, UK; 14 Wythenshawe Hospital, Manchester, UK; 15 St Thomas’ Hospital, London, UK; 16 Derriford Hospital, Plymouth, UK; 17 Sheffield Teaching Hospitals, Sheffield, UK; 18 Somerset Lung Centre, Musgrove Park Hospital, Taunton, UK; 19 Ninewells Hospital, Dundee, UK; 20 Royal Lancaster Infirmary, Morecambe, UK; 21 Royal Gwent Hospital, Newport, UK; 22 South Tyneside District Hospital, South Tyneside, UK; 23 Salford Royal Hospital, Salford, UK; 24 Glasgow Royal Infirmary, Glasgow, UK; 25 NHS Lanarkshire University Hospital Monklands, Airdrie, UK; 26 Glenfield Hospital, University Hospitals of Leicester NHS Trust, Leicester, UK; 27 NIHR Leicester Biomedical Research Centre Respiratory Diseases, Leicester, UK; 28 Churchill Hospital, Oxford Respiratory Trials Unit, Oxford, UK; 29 NIHR Oxford Biomedical Research Centre, Oxford, UK; 30 Kettering General Hospital, Kettering, UK; 31 Western General Hospital, Edinburgh, UK; 32 Freeman Hospital, Newcastle upon Tyne, UK; 33 Royal Papworth Hospital, Cambridge, UK; 34 Gartnavel General Hospital, Glasgow, UK; 35 Department of Imaging, Queen Elizabeth University Hospital, Glasgow, UK; 36 Laboratory of Experimental Medicine, University of Antwerp, Antwerpen, Belgium

**Keywords:** Mesothelioma, Pleural Disease, Asbestos Induced Lung Disease

## Abstract

**Introduction:**

Pleural mesothelioma (PM) is often presaged by benign asbestos-associated pleural inflammation (AAPI), offering a unique window of opportunity for translational research. The PREDICT-Meso International Accelerator Network is leveraging this natural history to perform target identification and develop novel therapies for early-stage or pre-invasive disease. This requires assembly of a unique bioresource of longitudinal human tissue samples spanning the terminal stages of PM evolution, development of preclinical models for drug screening and reliable tools for risk prediction in patients presenting with AAPI.

**Methods and analysis:**

Mesothelioma Observational study of Risk prediction and Generation of paired benign-meso tissue samples, Including a Nested MRI Substudy (Meso-ORIGINS) is a prospective, multicentre observational study, comprising two arms (A and B), with a nested MRI substudy in arm A. Arm A will recruit 300 AAPI patients and perform 6-monthly surveillance for 2 years. Suspicion of PM evolution will prompt repeat biopsy and banking, delivering a primary objective of ≥38 longitudinal AAPI-PM tissue pairs. This target reflects a projected PM evolution rate of 14% (95% CI 10.5 to 19.2) derived from a prior multicentre feasibility trial. Multiomic risk profiling will be performed in arm A, using blood proteomics, exhaled breath metabolomics and perfusion MRI. Arm B will recruit 300 patients with suspected PM, permitting collection of multiregion pleural biopsies in patients spanning AAPI and PM timepoints for evaluation of anatomical heterogeneity. Where possible, patients in arm B diagnosed with AAPI will be recruited to arm A for 2-year surveillance +/− repeat biopsy in subsequent PM evolution cases. Pleural fluid will be collected in arm B for cell-line generation and diagnostic biomarker evaluation. Exhaled breath will be collected in arm B for diagnostic biomarker evaluation.

**Ethics and dissemination:**

The study has ethical approval (REC Ref 21/WS/0120). Results will be disseminated via peer-reviewed journals and national/international scientific conferences. Tissues, data and derived omics will be shared via the PREDICT-Meso Research Tissue Bank (REC Ref 21/WS/0011).

**Trial registration number:**

ISRCTN22929761.

**WHAT IS ALREADY KNOWN ON THIS TOPIC**

- Approximately 15% of patients with benign asbestos-associated pleural effusion (also known as asbestos-associated non-specific pleuritis) are diagnosed with pleural mesothelioma (PM) within 2 years. This presents a unique opportunity for translational research focused on the development of novel therapies for use in early-stage or preinvasive disease.

**WHAT THIS STUDY ADDS**

- Mesothelioma Observational study of Risk prediction and Generation of paired benign-meso tissue samples, Including a Nested MRI Substudy (Meso-ORIGINS) is the prospective tissue banking and risk prediction vehicle for the PREDICT-Meso International Accelerator network, funded by Cancer Research UK and its partners in Italy and Spain. The study is recruiting 600 patients across two arms, with Arm A focused on longitudinal follow-up of 300 asbestos-associated pleural inflammation (AAPI) patients, with repeat biopsy at PM evolution. Arm B is focused on multiregion pleural biopsy collection for evaluation of anatomical heterogeneity. The study is open at 32 sites in 27 UK cities.

**HOW THIS STUDY MIGHT AFFECT RESEARCH, PRACTICE OR POLICY**

- The tissue resources generated are being used for target identification, preclinical model development and the screening and validation of new therapies ready for human trials. Multiomic risk prediction in arm A, including blood proteomics, exhaled breath metabolomics and perfusion MRI will inform the eligibility criteria for future precision prevention trials. The samples collected, including pleural tissue, pleural effusion, blood, imaging, clinical data and derived omics, are being shared with the global PM research community via a REC-approved research tissue bank.

## Introduction

 Pleural mesothelioma (PM) is an aggressive thoracic malignancy causally linked to prior asbestos exposure.^1^ The carcinogenic properties of asbestos were first established in the 1960s,^2^ eventually prompting import and utilisation bans in most developed countries, including the UK and EU. Unfortunately, asbestos continues to be used in other nations, including the USA, Brazil, Russia, India, China and Indonesia.^3^ Environmental exposures related to in-situ asbestos are also rising in countries with import bans, including the UK, reflecting the lack of safe removal strategies.^4^ Consequently, the WHO estimates that 125 million people are exposed to asbestos annually and the global incidence of PM is predicted to rise from 30 000 cases currently to >50 000 cases per year by 2040.^3^,^5^ Despite recent positive clinical trials in advanced PM, the disease remains universally fatal, with a median survival of 12–18 months.^6^,^8^ There is an urgent need for accelerated research, including the testing of novel or repurposed therapies in patients with earlier stages of disease, where no current standard of care exists.^6^,^8^

Salient molecular features of PM include a low mutational burden and a genomic landscape dominated by tumour-suppressor loss, with few oncogenic drivers.^9 10^ This poses obvious challenges for the development of effective drug therapies. However, PM is also preceded by decades of pleural inflammation with a typical latency of 30–60 years following asbestos exposure. Critically, this manifests as a clinically overt and symptomatic pleural effusion in some patients, offering a unique window of opportunity for longitudinal translational research.^10^,^13^ Apparently benign asbestos-associated pleural effusion has attracted various labels, including non-specific pleuritis (NSP), benign asbestos pleural effusion and asbestos-associated pleural inflammation (AAPI). In a recent meta-analysis of 17 studies, including 2607 NSP cases, PM evolution occurred in 5.44% (95% CI 3.37 to 7.51).^14^ However, PM evolution was much more common in asbestos-exposed NSP or AAPI: 14.9% (95% CI 10.94 to 18.85).^14^ It is currently unclear whether AAPI represents a genuine PM precursor lesion, or false-negative sampling in a patient with unmeasurable early-stage PM somewhere in the pleural space. The recent characterisation of mesothelioma in situ,^15^ supports the existence of a transitional state in all patients. The PREDICT-Meso International Accelerator Network has been established to leverage the natural occurrence of AAPI and assemble unique longitudinal pleural tissue samples for translational research, specifically for target identification, preclinical model development and drug screening of new therapies suitable for early-stage or preinvasive disease. The project is funded by Cancer Research UK (CRUK), Fundación Científica de la Asociación Española Contra el Cáncer (FC AECC) and the Fondazione Associazione italiana per la Ricerca sul Cancro and currently involves 175 investigators from 101 institutions in 17 countries. The Mesothelioma Observational study of Risk prediction and Generation of paired benign-meso tissue samples, Including a Nested MRI Substudy (Meso-ORIGINS) study described here constitutes the prospective tissue collection vehicle within the PREDICT-Meso programme. The tissue and data collected feed downstream work packages, which also address risk prediction and the selection of suitable patients for future precision prevention/early-stage therapeutic trials. Evolving data concerning the marked spatial (intertumour, intrapatient) heterogeneity of PM supports a discovery strategy that integrates data from multiple biopsy sites.^16^,^18^ Meso-ORIGINS is, therefore, also collecting multiregion thoracoscopic biopsies. The final design of the study, including the eligibility criteria deployed and sample size were established from data collected in the previously published, mixed methods, multicentre Meso-ORIGINS feasibility study.^11^

## Methods and analysis

### Study design and setting

**Figure 1:**
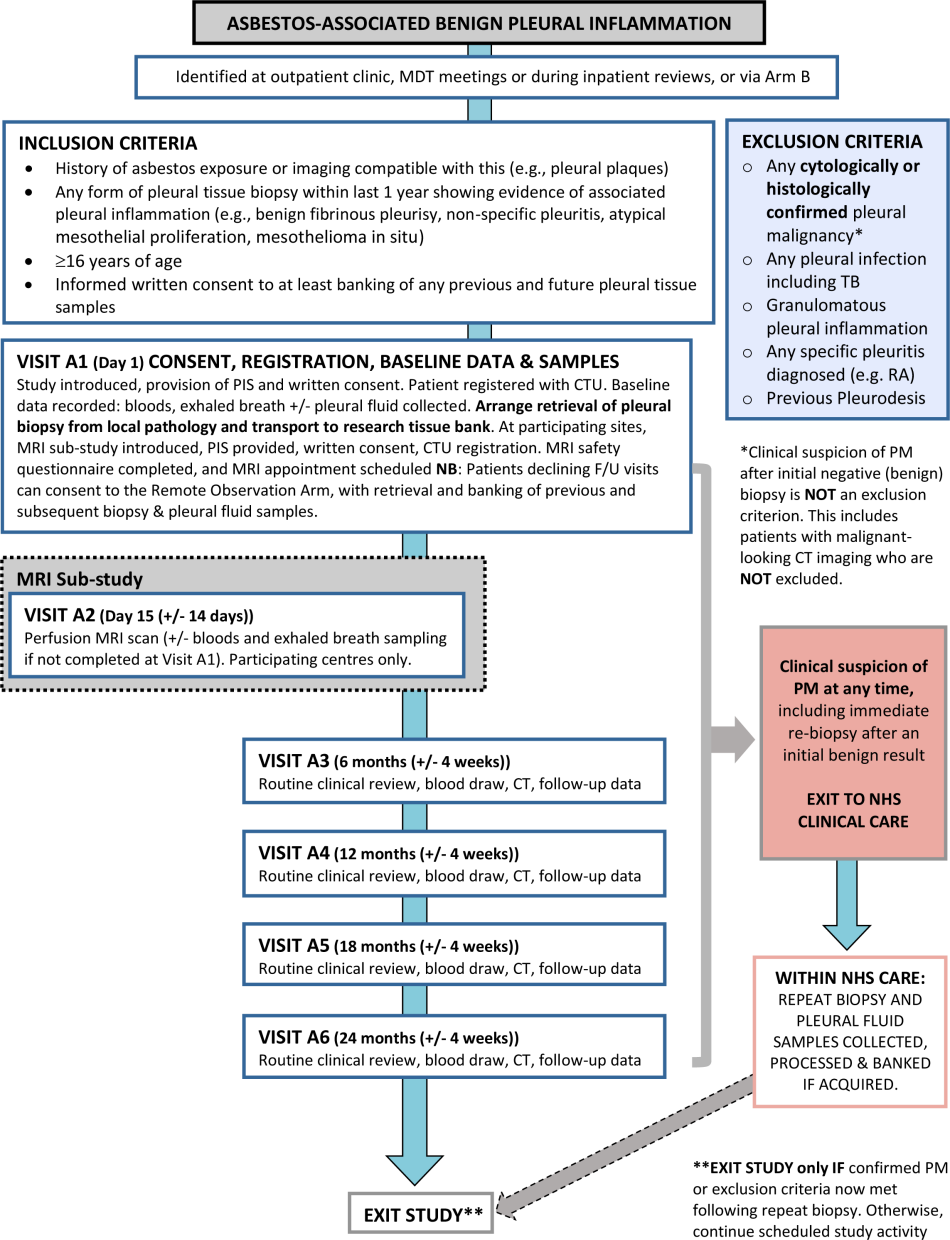
Meso-ORIGINS arm A eligibility criteria and study visit flowchart. CTU, Clinical Trials Unit; F/U, follow-up; Meso-ORIGINS, Mesothelioma Observational study of Risk prediction and Generation of paired benign-meso tissue samples, Including a Nested MRI Substudy; MDT, multidisciplinary team; PIS, participant information sheet; PM, pleural mesothelioma; RA, rheumatoid arthritis; TB, tuberculosis.

Meso-ORIGINS is a multicentre, prospective, longitudinal observational study, incorporating two arms, arm A and arm B, and a nested MRI substudy within arm A. The overall design is summarised in figure 1 (arm A) and 2 (arm B).

### Study objectives and endpoints

Objectives and their associated endpoints are summarised in table 1.

**Table 1:** Study objectives and associated endpoints

	Objective	Associated endpoint
Primary	To create a prospective cohort of patients with asbestos associated benign pleural disease of whom an estimated 38 will develop PM within 2 years	Number of patients in arm A diagnosed with PM at any point from study registration to completion of 2 years follow-up
Secondary	To generate a risk prediction model for evolution of PM within 2 years, based on serum proteomics, exhaled breath metabolomics and perfusion MRI	Results of a multiomic risk classifier based on radiomic, proteomic and metabolic measurements in patients at baseline in arm A
	To collect spatially distinct tumour biopsies from patients with PM, facilitating comprehensive characterisation of intrapatient tumour heterogeneity	Number of patients in arm B with histologically confirmed PM following thoracoscopy
	To determine the diagnostic performance of an exhaled breath diagnostic classifier for detection of PM prior to thoracoscopy	Sensitivity and specificity of an exhaled breath diagnostic classifier for histologically confirmed PM based on samples collected prior to thoracoscopy

PM, pleural mesothelioma.

### Eligibility assessment

#### Arm A

Arm A will recruit asbestos-exposed patients with a histological diagnosis of AAPI (via any pleural biopsy method). A persisting clinical suspicion of PM after an initial benign pleural biopsy is not an exclusion criterion and such participants will be eligible, even if immediate rebiopsy is planned. This includes participants with malignant-looking CT imaging (eg, pleural thickening >1 cm, pleural nodules, fissural nodules). All patients will be subjected to the following eligibility criteria:

##### Inclusion criteria

- History of asbestos exposure or imaging compatible with this (eg, pleural plaques).
- Any form of pleural tissue biopsy within the last 1 year showing evidence of associated pleural inflammation (eg, benign fibrinous pleurisy, NSP, atypical mesothelial proliferation, mesothelioma in situ).
- ≥16 years of age.
- Informed written consent to at least banking of previous and future pleural tissue samples

##### Exclusion criteria

- Any cytologically or histologically confirmed PM.
- Any pleural infection, including tuberculosis.
- Granulomatous pleural inflammation.
- Any specific pleuritis diagnosed (eg, rheumatoid arthritis).
- Previous pleurodesis.

### Arm B

Arm B will recruit asbestos-exposed participants undergoing thoracoscopy with a clinical suspicion of PM. All patients will be subjected to the following eligibility criteria:

#### Inclusion criteria

- Suspected PM, as defined by a unilateral pleural effusion or pleural-based mass lesion.
- History of asbestos exposure or imaging compatible with this (eg, pleural plaques).
- Sufficient fitness for thoracoscopy (local anaesthetic thoracoscopy (LAT) or video-assisted thoracoscopic surgical (VATS) are permissible).
- ≥16 years of age.
- Informed written consent.

#### Exclusion criteria

- Current or recent (within the last 3 months) intercostal chest drain.
- Previous pleurodesis

### Study setting

Recruitment began in July 2022, with a 49-month recruitment period planned. A minimum of 25 sites were planned based on recruitment numbers and rate in the preceding feasibility study.^11^ To qualify, centres are required active pleural diagnostic services, including thoracoscopy for arm B involvement. At the time of writing, 32 sites are open across 27 cities (see online supplemental table S1).

### Identification of participants and consent

Potential participants will be identified through clinics, inpatient reviews and/or multidisciplinary team (MDT) meetings. It is anticipated that arm B cases will primarily be identified via urgent suspicion of cancer or 2-week wait urgent referral pathways. Potential participants will be screened by trained members on the delegation log, and eligibility will be confirmed by the site principal investigator before enrolment. Eligible participants will be provided with a participant information sheet (PIS) and given sufficient time, in their judgement, to consider involvement. Same-day consent is permissible in participants who are comfortable with this. Participants who would like more time to consider will be contacted no later than 2 working days following the provision of a PIS. Participants will be made fully aware that enrolment is voluntary and will not influence routine care. Where enrolment is agreed, written informed consent will be obtained and the participant will be registered with the Glasgow Oncology Clinical Trials Unit (GO CTU). Consent will be sought to obtain blood, breath, pleural fluid and pleural tissue samples and for these samples to be stored indefinitely in the REC-approved PREDICT-Meso Research Tissue Bank (RTB) (21/WS/0011) for use in future ethically approved projects. Participants will also be asked to provide consent for anonymised clinical and imaging data to be used in future related and ethically approved research. Arm B patients with a subsequent diagnosis of AAPI may be eligible for transition to arm A, facilitating subsequent surveillance±-rebiopsy if PM evolution is suspected. This will require separate arm A PIS, consent and registration forms and a separate study ID.

### Study procedures

#### Visit schedules by arm

In arm A, following a baseline assessment (visit A1), study follow-up will occur at 6-monthly intervals (±4 weeks) for 2 years (visits A3–A6) and will preferably align with routine clinical care, see figure 1. Patients enrolled in the MRI substudy will complete visit A2 within 15 (±14) days of baseline assessment. Arm A participants who opt for remote observation (RO) are not required to attend follow-up study visits A3–A6. Site teams will instead conduct remote surveillance using electronic health records and/or via liaison with the clinical team.

**Figure 2:**
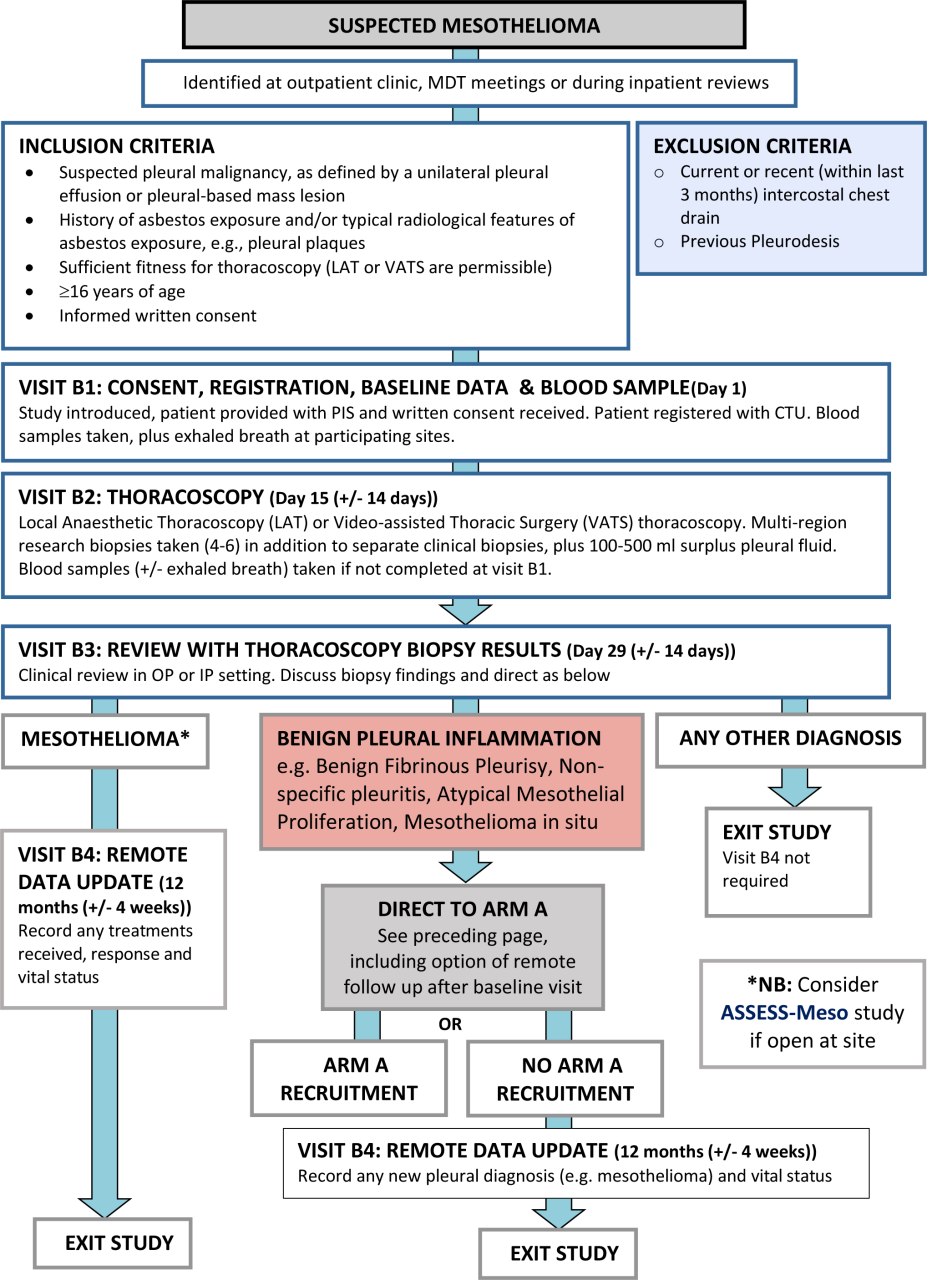
Meso-ORIGINS arm B eligibility criteria and study visit flowchart. CTU, Clinical Trials Unit; IP, Inpatient; Meso-ORIGINS, Mesothelioma Observational study of Risk prediction and Generation of paired benign-meso tissue samples, Including a Nested MRI Substudy; MDT, multidisciplinary team; OP, Outpatient; PIS, participant information sheet.

In arm B, following a baseline assessment (visit B1), participants will have multiregion pleural biopsies and pleural effusion samples collected during thoracoscopy (visit B2) at day 15 (±14 days), before returning for clinic review and biopsy results (visit B3) at day 29 (±14 days), see figure 2. An additional remote visit will be required at 12 months (visit B4) in some patients, including arm B patients diagnosed with PM at visit B3, and patients diagnosed with AAPI at visit B3 who did not subsequently enrol into arm A.

#### Database

Electronic case report forms (eCRF) will be used to collect data at baseline and all follow-up visits. eCRFs will be saved in a MACRO database (V.4.11.0.1238, 2026 Ennov). Data collection will be performed at least every 6 months following registration for arm A participants who opt for RO, ensuring data alignment across Arm A.

#### Baseline data collection

At baseline assessment in both study arms (visit A1 and B1), the data collected includes patient demographics, asbestos exposure history, presenting symptoms, performance status (PS), comorbidities and mode of presentation. Baseline blood and pleural fluid results can be extracted from routine clinical records if measured within 4 weeks of the study visit. Required data include full blood count, urea and electrolytes, C reactive protein, albumin, serum and effusion total protein, serum and effusion lactate dehydrogenase, plus effusion glucose, microbiology and cytology. Where these data are not available within this window, blood tests will be repeated at the same time as research bloods (see the section Translational bloods). In arm A, details of the biopsy on which the AAPI diagnosis was made will also be collected, including biopsy date, biopsy method and histological descriptors. Results of contrast-enhanced CT thorax should also be recorded at baseline, ideally acquired within 12 weeks of visit A1/B1.

#### Tissue retrieval and sampling

##### Arm A

At baseline assessment (visit A1), study teams will initiate biopsy retrieval. This will require liaison with their local pathology department for labelling and shipment of all archival Formalin Fixed Paraffin Embedded (FFPE) blocks corresponding to the index diagnosis of AAPI. This process will be repeated for any follow-up biopsies performed in the study period with suspicion of PM evolution.

##### Arm B

In arm B, dedicated multiregion biopsies (MRBs) will be collected at thoracoscopy (Visit B2, Day 15±14 days), which can be LAT or VATS thoracoscopy. Research samples will be taken only after all clinical biopsies have been completed. MRBs will be taken from up to six sites, ideally from a variety of anatomical locations, which will be mapped using the Thoracoscopy Worksheet (see online supplemental figure S2). The number and location of MRB sampling will be at the operator’s discretion, factoring patient tolerance (in conscious LAT patients) and the distribution of visible abnormalities. Research samples will be placed in individual formalin-filled biopsy pots. A separate FFPE block will then be generated for each biopsy site, performed by local pathology departments following standard laboratory processes.

##### Tissue banking

Archival clinical (Arm A) and multi-region research (arm B) FFPE blocks will be transported to the REC-approved PREDICT-Meso Research Tissue Bank (RTB, REC Ref 21/WS/0011), which is a satellite tissue bank associated with the NHS Greater Glasgow and Clyde Biorepository, based at the Queen Elizabeth University Hospital, Glasgow. The processes for transport to this site are described in a dedicated Meso-ORIGINS Sample Handling Manual and Biopsy Manual (available at https://www.predictmeso.com/results-and-outputs/).

### Follow-up visits

#### Arm A

##### 6-monthly follow-up (visits A3-A6)

Follow-up outpatient reviews will be performed at 6-monthly intervals (±4 weeks) (visits A3–A6). Ideally, these visits will be combined with routine clinical follow-up to minimise the burden on patients and staff. Vital status, physical characteristics (weight, PS, MRC dyspnoea score) and the results of any updated imaging will be recorded. The explicit clinical opinion of the PI or delegate, regarding any suspicion of PM evolution will also be recorded. A remote follow-up option is available for participants who cannot return for face-to-face follow-up visits. Where this option is used, research teams are required to perform adequate surveillance, ensuring any naturally occurring PM evolution is identified promptly.

##### Repeat biopsy if mesothelioma evolution suspected

If a clinical suspicion of PM evolution is recorded at visit A3–A6, repeat pleural biopsies will be performed at the discretion of the clinical team. This action can also be taken at any naturally occurring timepoint between 6-month study visits, for example, if a participant presents with progressive pleural effusion, evolving pleural nodularity, constitutional symptoms, chest wall pain and/or other new features. If a repeat pleural biopsy is performed, the biopsy date, method and results will be captured, including histological subtype and stage of any confirmed PM evolutions. This will include postmortem biopsies, which are routinely required in patients who die with a suspicion of PM that could not be confirmed in life. FFPE blocks corresponding to the repeat biopsy will be banked via the processes outlined above (see the section Tissue banking).

##### Follow-up imaging

Contrast-enhanced CT thorax should ideally be acquired within 12 weeks of visits A3–A6. However, this is not mandatory. In some centres, patients will have six monthly chest radiographs, with additional CT surveillance based on clinical judgement.

### Arm B

Follow-up after LAT/VATS will occur at visit B3 (Day 29±14 days). Biopsy results will be recorded including histological subtype and stage in PM cases. At 12 months, a remote data update will be completed (visit B4) in PM patients to capture vital status, treatments received and response. Visit B4 will also apply to patients diagnosed with AAPI at visit B3, unless that patient was subsequently recruited to arm A. This will allow recording of vital status and any PM evolution in these participants. Patients with any other pleural diagnosis recorded at visit B3 (eg, metastatic PM) will exit the study after visit B3.

### Translational bloods

Research blood samples for risk prediction modelling and early detection of PM evolution will be drawn at baseline, and all follow-up visits in arm A (see online supplemental tables S3 and S4). Baseline (A1 and B1) and follow-up (A3–A6) blood samples will be collected and stored as whole blood, plasma and serum. Blood for germline DNA will be collected at A1 and B1 only. Sample processing will be standardised, as detailed in the Meso-ORIGINS Sample Handling Manual (available at https://www.predictmeso.com/results-and-outputs/), with all samples frozen at −80 within 2 hours.

### Exhaled breath sampling

At participating sites, exhaled breath samples will be collected at baseline in both arms (visits A1 and B1). The utility of previously reported volatile organic compound (VOC) biomarkers will be tested using these samples for risk prediction and non-invasive detection of PM in arms A and B, respectively.^19 20^ Detailed instructions are provided in the Meso-ORIGINS Exhaled Breath Manual (available at https://www.predictmeso.com/results-and-outputs/).

### Pleural fluid collection

Pleural fluid will be collected in both arms for several purposes, including proteomic analyses for risk prediction in arm A and cell line generation in arm B. At baseline in arm A, pleural fluid will frequently not be available as AAPI patients rarely have an actively draining effusion. However, an effusion sample will be collected at visit A1 if patients have an indwelling pleural catheter in situ. Pleural fluid will also be acquired during any follow-up biopsy procedures in arm A, where possible. In arm B, pleural fluid will be acquired at the time of thoracoscopy (visit B2) alongside MRBs. Pleural fluid processing will be standardised with different processes required for different purposes (see Meso-ORIGINS Sample Handling Manual, available at https://www.predictmeso.com/results-and-outputs/). Samples for cell line generation will be posted ambient and unprocessed to downstream labs in Glasgow, Cambridge and Oxford. Samples for biomarker analyses (eg, proteomics) will be processed with pleural pellet and supernatant stored at −80 within 2 hours.

### MRI sub-study in arm A

Contrast-enhanced MRI will be performed in arm A patients at sites participating in the MRI substudy, at visit A2. The objective of this substudy is to test the predictive value of previously reported perfusion MRI biomarkers, including MRI Early Contrast Enhancement.^21^ All participants will complete an MRI safety questionnaire+/−an orbital radiograph if a metallic foreign body is suspected. The MRI acquisition will be standardised as specified in the MRI manual (available at https://www.predictmeso.com/results-and-outputs/). Deidentified DICOM images, with accompanying metadata, will be securely transferred to the host centre for analysis.

### NIHR associate PI scheme

Meso-ORIGINS is enrolled on the NIHR Associate Principal Investigator Scheme, providing opportunities for early career researchers, with eight individuals involved to date.

### Statistical considerations

#### Sample size

The sample size estimate for arm A is 300, based on the outcome of the retrospective arm of the Meso-ORIGINS feasibility study and using prediction intervals (PI) for binomial data, as proposed by Lu and Jin.^22^ In this retrospective multicentre cohort study, PM evolution following an initial diagnosis of AAPI was confirmed histologically in 36/257 (14%, 95% CI (10.3 to 18.8)).^11^ Recruitment of 300 cases to Meso-ORIGINS will, therefore, generate 38 (95% PI (41 to 89)) biopsy-confirmed PM evolutions, assuming 10% loss to follow-up.

The MRI substudy will recruit at least 50 participants. This sample size reflects the availability of research MRI in the UK pleural disease network, based on experience in recent studies and is expected to generate approximately 6 cases of PM, assuming 10% loss to follow-up, and the evolution rate in the substudy tracks the rate in Arm A overall.

The sample size estimate for arm B is 300, which is expected to generate at least 109 PM cases (one-sided 95% PI (109,+ve Inf)) based on a study by Tsim *et al* in which 69/155 (44.5%) participants with asbestos exposure and a clinical suspicion of PM had diagnosis confirmed at thoracoscopy.^23^ The sample size of arm B was increased from an original arbitrary estimate of 39 and subsequent increases to 120 and 250 following encouraging early recruitment. These changes followed publication of a study by Zhang *et al,*^18^ which reported highly complex exomic intratumour heterogeneity (ITH) based on 90 multiregion pleural biopsies collected from 22 PM patients and increased downstream requirements for the tissue and data being generated. The larger sample size also afforded an opportunity to test the diagnostic utility of an exhaled breath classifier for PM.^19^ An interim review of recruitment in March 2025 revealed 181 arm B recruits, recruited over 33 months, with a crude average of 5.5 cases/month. We conservatively predicted recruitment of 7 cases/month over the remaining recruitment period (April 2025 to August 2026), translating into an additional 119 recruits and a total of 300 cases by study end. The larger number of confirmed PM cases (n=109) predicted from this cohort will generate an estimated 436 multiregion PM biopsies (assuming four biopsies/case), increasing the pool of samples for ITH assessment by 7.6-fold. We predict collection of exhaled breath in 70 of 119 (around 60%) additional arm B participants recruited. Assuming 90% sensitivity and 80% specificity of the final classifier, 44.5% prevalence of PM and a 5% type I error rate, 70 cases will deliver precision in the final estimates of sensitivity and specificity not exceeding 11%.

#### Patient and public involvement

The study management group includes a patient and public involvement (PPI) representative, who also contributed to protocol and patient-facing document design. They also contribute to ongoing reviews of study progress and emerging data.

### Statistical analysis plan

#### Primary endpoint analysis

The primary endpoint will be reported by simple descriptive statistics. The CI will be based on the Agresti-Coull approach.^24^

#### Secondary endpoint analyses

A classification model for PM evolution will be generated using the XGBoost tree boosting system, where SHAP values will be used to evaluate the contribution of each feature to individual predictions. Candidate features will include clinical variables and those derived from blood biomarker analyses (eg, proteomics), exhaled breath VOCs and perfusion MRI. The performance of the developed model will be assessed using a Receiver Operator Characteristic curve. Any model developed will be subjected to subsequent validation in an independent data set. The number of participants in arm B with confirmed PM following thoracoscopy will be reported by simple descriptive statistics. The CI will be based on the Agresti-Coull approach.^24^ The downstream heterogeneity analyses will be performed by a Bioinformatics team within the CRUK Scotland Institute, Glasgow and reported separately. The performance of the PM diagnostic classifier will be analysed using 2×2 contingency tables and reported as sensitivity and specificity, with 95% CIs.

### Changes to protocol

The text herein describes the current protocol (V.2.2, 8 May 2025). The following changes were made in previous versions, excluding V.1.1, 1.4, 1.5 and 1.7, which involved only grammatical changes:

V.1.2, dated 17 December 2021

- Updated lower age limit on eligibility, from 18 to 16, based on recent NIHR guidance.

V.1.3, dated 15 March 2022

- Clarification that germline blood should be collected in arm B and blood samples not collected at visit B1 could alternatively be collected at visit B2.

V.1.6, dated 22 August 2022

- Clarification that the PIS can be sent to potentially eligible patients ahead of clinic, where deemed clinically appropriate.

V.1.8, dated 21 March 2023

- Updated arm B target from 39 to 120 in response to positive early recruitment and emerging data regarding the role of spatial heterogeneity.
- Reduced MRI substudy target from 250 to 100 in response to MRI availability.
- Amendment to arm A follow-up visit timelines to accommodate local practices by extending visit window to 6 months ±4 weeks.

V.1.9, dated 11 August 2023

- Addition of remote consent in arm A to optimise patient access following feedback from several sites that cover large geographical areas.

V.2.0, dated 15 January 2024

- Updated arm B target from 120 to 250 in response to positive recruitment and good transition of participants with AAPI into arm A for longitudinal follow-up.
- Optimisation of arm B translational sampling to include the collection of serum and plasma in addition to germline blood. The volume of pleural fluid banked was increased to 240 mL in response to downstream pipeline requirements.

V.2.1, dated 14 September 2024

- Removal of arm A visits A7–A9, which involved repeat (research only) biopsy in patients without PM evolution after at least 18 months follow-up. After review of the first 20 patients and discussion with sites, this activity was deemed unfeasible.
- Reduced arm A target from 500 to 300 due to slower-than-anticipated site set-up.

V.2.2, dated 8 May 2025

- Updated timelines to extend recruitment by 1 year with a recruitment end date of 31 August 2026 and new study end date of 31 August 2028.
- Addition of exhaled breath collection to arm B to address the new secondary objective ‘To determine the diagnostic performance of an exhaled breath diagnostic classifier for detection of PM prior to thoracoscopy’.
- Addition of 1-year remote follow-up in arm B (visit B4).
- Updated arm B target from 250 to 300 in response to updated study timelines.
- Retrieval of postmortem biopsies performed as part of routine care within arm A.
- Removal of remote consent option for RO participants, minimising numbers without germline blood samples for downstream genomic analyses.

### Definition of end of study

The study will end on the date of last data capture following accrual of target recruitment numbers (n=300) for arms A and B separately.

## Ethics and dissemination

### Ethical approval

The original study protocol and all subsequent amendments have been approved by the West of Scotland Research Ethics Committee 1 (21/WS/0120).

### Safety considerations

All adverse events (AEs) and serious adverse events (SAEs) thought to be related to study procedures will be recorded. This includes AEs resulting from blood sampling, breath collection, MRI and thoracoscopy (LAT or VATS). For participants in the MRI substudy, AEs and SAEs thought to be related to the MRI acquisition, including administration of gadolinium contrast, or the X-ray of orbits (if required) will also be recorded. Safety reporting is overseen by the Pharmacovigilance Department of the GO CTU as delegated by the trial Sponsor.

### Dissemination

Study results will be presented at national and international scientific meetings and published in full in a peer-reviewed journal. All study outputs will also be shared via the PREDICT-Meso webpage and social media channels.

### Study management

Meso-ORIGINS will be coordinated by the GO CTU working in close collaboration with the PREDICT-Meso Project Manager. The SMG, comprising the chief investigator, selected coinvestigators, project manager, statistician, trial coordinator, pharmacovigilance coordinator, PPI representative and IT programmer meet monthly to oversee the study.

## Supplementary material

10.1136/bmjresp-2025-004045 online supplemental file 1

## Data Availability

Data are available upon reasonable request.
