# Supplementary material for: Protocol for Mesothelioma Observational study of Risk prediction and Generation of paired benign-meso tissue samples, Including a Nested MRI Substudy (Meso-ORIGINS)
Source: BMJ Open Respir Res. 2026 Jul 1;13(1):e004045. doi: 10.1136/bmjresp-2025-004045 (PMC13331108; doi:10.1136/bmjresp-2025-004045)
Supplement: online supplemental file 1 [file bmjresp-13-1-s001.pdf]

Protocol for Mesothelioma Observational study of Risk prediction and Generation of paired benign-meso tissue samples, Including a Nested MRI Sub-study (Meso-ORIGINS)

ONLINE ONLY SUPPLEMENT

Contents

Section 1	Supplementary Table S1: Recruiting Centres	Page 1
Section 2	Supplementary Figure S2: Meso-ORIGINS Biopsy Worksheet	Page 2
Section 3	Supplementary Table S3: Arm A Study Visit Schedule	Page 4
Section 4	Supplementary Table S4: Arm B Study Visit Schedule	Page 6

SECTION 1: Supplementary Table S1 - Recruiting Sites

Geographical Site	Hospital(s)	Date Opened
Aberdeen	Aberdeen Royal Infirmary	15-12-22
Blackpool	Blackpool Teaching Hospital	24-03-23
Bristol	Southmead Hospital	16-06-22
Cambridge	Royal Papworth Hospital	09-11-23
Clydebank	Golden Jubilee National Hospital	16-06-22
Devon	Royal Devon and Exeter Hospital	30-06-25
Dundee	Ninewells Hospital	30-11-23
Edinburgh	Western General Hospital	02-08-23
Glasgow	Queen Elizabeth University Hospital<br>Glasgow Royal Infirmary<br>Gartnavel General Hospital	16-06-22
Inverness	Raigmore Hospital	28-11-23
Kettering	Kettering General Hospital	15-03-23
Lanarkshire	Monklands General Hospital	19-03-25
Leicester	Glenfield Hospital	14-11-22
London	St Thomas' Hospital	12-11-25
Manchester	Wythenshawe Hospital	10-10-22
Morecambe	Royal Lancaster Infirmary<br>Furness General Hospital	02-08-22
Newcastle	Freeman Hospital	16-02-24
Newport	Royal Gwent Hospital<br>St Woolos Hospital<br>Grange University Hospital	19-07-22
Stockton	University Hospital of North-Tees	28-03-24
Northumbria	Northumbria Specialist Emergency Care Hospital	26-07-22
Oxford	Churchill Hospital	05-08-22
Plymouth	Derriford Hospital	10-10-22
Salford	Salford Royal Hospital	20-09-22
Sheffield	Northern General Hospital	18-11-24
South-Tyneside	South Tyneside District Hospital	22-05-24
Stoke	Royal Stoke University Hospital	15-01-25
Taunton	Somerset Lung Centre	25-07-22

SECTION 2: Supplementary Figure S2 - Meso-ORIGINS Biopsy Worksheet

MesO-ORIGINS	**MESO-ORIGINS**<br>**THORACOSCOPY WORKSHEET: Arm B, Visit B2**

Meso-ORIGINS: **Meso**thelioma **O**bservational study of **RI**sk prediction and **G**eneration of paired benign-meso tissue samples, **I**ncluding a **N**ested MRI **S**ub-study

**Patient Initials:** (F) ____ (S) ____	**Date of Birth:** DD / MON / YYYY	**Study Number:**

GENERAL

Side: o Right o Left **Procedure Type** o LAT o VATS

Septations: o Yes o No **Volume drained:**______________ **ml**

PROCEDURE DETAILS

**DRUG**	**OPTION (please ü)**	**DOSE**
**Pre-medication**	o **Oramorph**	________**mg**
	o **Atropine**	________**mg**
	☐ **Sevredol**	________**mg**
	☐ **Other, specify *(incl unit)*:** ____________________	________
**Sedation**	☐ **Midazolam**	________**mg**
	☐ **Propofol**	________**mg**
	☐ **Other, specify *(incl unit)*:** ____________________	
	☐ **General Anesthesia**	
**Local anaesthetic**	☐ **Lidocaine** ☐ **1%** ☐ **2%** ________**ml**	☐ **Adrenaline inclusion**
	☐ **Other, specify *(incl unit)*:** ____________________	________
**Analgesia**	☐ **Alfentanyl**	________**micrograms**
	☐ **Fentanyl**	________**micrograms**
	☐ **Morphine**	________**mg**
	☐ **Other, specify *(incl unit)*:** ____________________	________

US on table: ☐ Yes ☐ No **Boutin with US:** ☐ Yes ☐ No

Fluid on US: ☐ Yes ☐ No ☐ N/A **Talc:** ☐ Yes ☐ No **If yes, dose given:**________ g

Boutin induction: ☐ Yes ☐ No **Drain size:** _____**F**

IMMEDIATE COMPLICATIONS: IF NONE TICK HERE ☐

Haemorrhage requiring transfusion: ☐ Yes ☐ No **Failure of procedure:** ☐ Yes ☐ No

Hypotension requiring intervention: ☐ Yes ☐ No **Other** ☐ **Yes specify:**______________________________

BIOPSY DETAILS

RESEARCH BIOPSIES<br>Ideally 4-6 from different zones			CLINICAL BIOPSIES	
**POT**	**ZONE**	**APPEARANCE**	**ZONE**	**APPEARANCE**
**#1**				
**#2**				
**#3**				
**#4**				
**#5**				
**#6**				

RECORD APPEARANCE IN ZONES AS:

- MACRO - macroscopic nodules
- MICRO - microscopic nodules
- THICK - pleural thickening
- PLAQUE – calcified pleural plaque
- NORMAL – no abnormality seen

BIOPSY SAMPLING

- See Biopsy Manual Section 7.1.7

RESEARCH BIOPSIES:

- Six pots are provided in the visit pack.
- Label the biopsy pots #1, #2, #3, #4, #5, #6.
  Along with Study ID and Visit (e.g. MO-001, B2, #4)
- **Samples from a single biopsy site must be put in a single numbered biopsy pot.**
- Multiple biopsies might be taken from the **same site** to get deeper biopsies - use the **same pot**.
- If biopsies are taken from a **different site** in the same zone - use a **different pot**.
- **Biopsies from different sites should NEVER GO IN THE SAME POT.**
- Record the zone and appearance of each biopsy site

CLINICAL BIOPSIES:

- Clinical biopsies should be sent in a **separate biopsy pot** (not provided) for **normal diagnostic processing**
- Number of clinical biopsies at operator discretion.

NOTES ON PLEURAL ZONE MAP BELOW: Odd-numbered parietal zones correspond to upper half of field of view. The dashed line = the visceral surface of the lung. Zone 11=Diaphragm. Zones 12-14=RLL, RML & RUL on right; LLL, Lingula & LUL on left

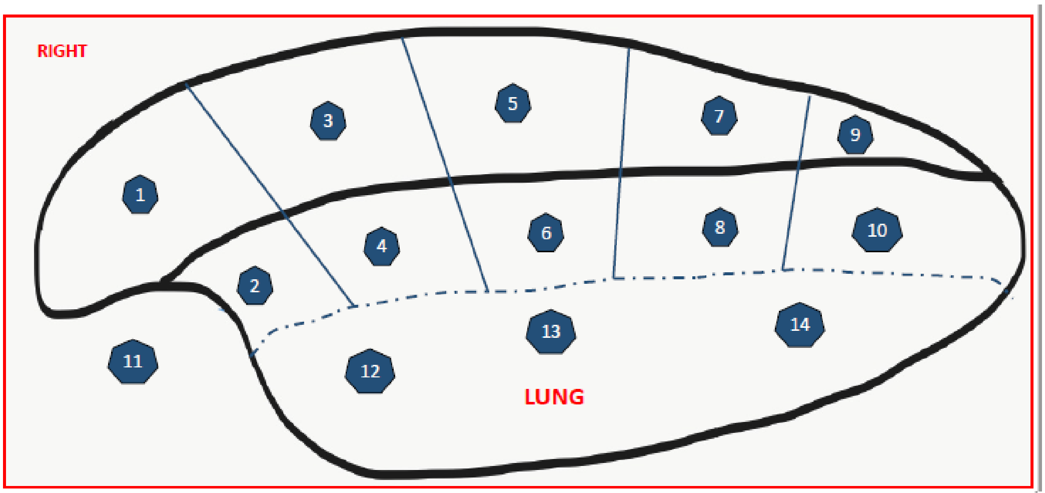

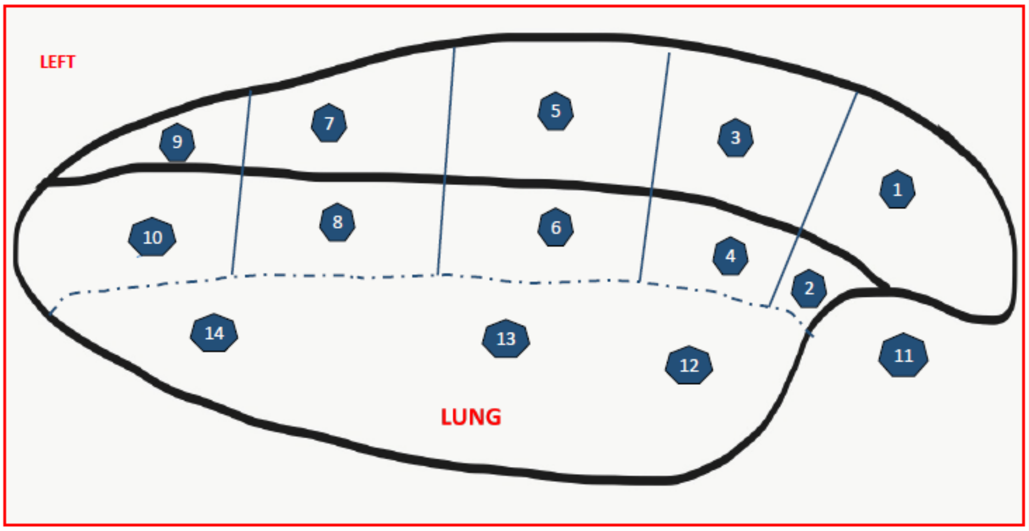

SECTION 3: Supplementary Table S3 - Arm A Visit Schedule

Visit Number	A1	A2	A3	A4	A5	A6
Approximate Study Day	1[1]	15d (± 14d)	6m (±4w)	12m (±4w)	18m (±4w)	24m (±4w)
**Routine Clinical Activity**						
Clinical review	X		X	X	X	X
Contrast-enhanced CT Thorax[2]	X		X	X	X	X
Arrange repeat imaging +/- biopsy if clinically indicated (suspected progression to PM)[3]	X		X	X	X	X
**Study Activity**						
Review Eligibility Criteria	X					
If potentially eligible, introduce study[4], provide with PIS and discuss participation[5]	X					
Informed Written Consent	X	X[6]				
Register participant with CTU	X	X[6]				
Record Baseline Data[7]	X	X[6]				
Blood Sampling, Processing and Banking[8]	X	X[6]	X[9]	X[9]	X[9]	X[9]
Exhaled Breath Sampling and Processing[10]	X	X[6]				
Bank Pleural Fluid sample if IPC in-situ[11]	X	X[6]				
Arrange retrieval of FFPE pleural tissue biopsies from local pathology and transport to RTB	X	X[6, 12]				
Bank any repeat pleural biopsies +/- fluid if **CLINICAL SUSPICION OF MESOTHELIOMA**	At any time from registration to study exit[11]					
Record Follow-up Data			X	X	X	X
Record Adverse Events	X	X	X	X	X	X
**MRI Sub-study Activity[13]**						
Review Eligibility Criteria	X					
Introduce MRI sub-study if eligible	X					
Provide separate MRI sub-study PIS	X					
Discussion and Written Informed Consent	X	X[6]				
Register participant to sub-study with CTU	X	X[6]				
Arrange a date for MRI	X					
MRI Safety Questionnaire	X	X[6]				
Orbital Radiograph, if indicated[14]		X				
Contrast-enhanced MRI Thorax[15]		X				

1. Visit A1 activities should ideally be completed on the same day but can be completed over up to 7d (see d)
2. A baseline contrast-enhanced CT Thorax should ideally be available within 12 weeks of visit A1 to confirm participant has not progressed since original benign biopsy. If not available, repeat CT should be considered based on clinical judgement but is not mandatory
3. Please refer to Meso-ORIGINS Biopsy Manual
4. Investigators may introduce the study at earlier clinic visits if eligibility likely and clinically appropriate

5. PIS can be provided either in person or remotely. Participants will be offered a follow-up telephone call with a member of the study team if they wish more time to consider the study. This call will occur no later than 2 working days after provision of PIS.
6. If not already performed
7. Including the following baseline blood results from patient records, which should be repeated if not available within 4 weeks of visit A1: full blood count, lactate dehydrogenase, c-reactive protein, albumin, urea and electrolytes. Baseline data also includes results of previous pleural fluid, pleural biopsy and imaging tests performed as part of routine clinical care.
8. Please refer to Meso-ORIGINS Sample Handling Manual
9. Follow-up visits are not required in Remote Observation cases but remote review and 6 monthly data updates, including vital status and any repeat pleural biopsies are required in all participants
10. Please refer to Meso-ORIGINS Exhaled Breath Sampling Manual. Exhaled breath samples can be omitted at sites where facilities are not in place for acquisition or storage, or on grounds of patient preference
11. It is acknowledged that not all patients will have pleural fluid available for banking, via an indwelling pleural catheter (IPC). Where available a sample should be drawn and banked. Please refer to the Meso-ORIGINS Sample Handling Manual for detailed instructions
12. FFPE biopsy blocks from diagnosis of BENIGN PLEURAL INFLAMMATION and histological confirmation of MESOTHELIOMA EVOLUTION or NO MESOTHELIOMA EVOLUTION should all be retrieved from the local pathology archive and transported to the PREDICT-Meso Research Tissue Bank (RTB), based in Glasgow. Please refer to the Meso-ORIGINS Sample Handling Manual for detailed instructions. Note that relevant repeat pleural biopsies include samples taken post-mortem. These samples should therefore be retrieved
13. Only offered to participants in participating centres
14. Orbital Radiograph only if required to exclude a foreign body, based on relevant history
15. Please refer to Meso-ORIGINS MRI Manual

SECTION 4: Supplementary Table S4 - Arm B Visit Schedule

Visit Number	B1	B2	B3	B4
Approximate Study Day	1	15d (±14d)	29d (±14d)	12m (±4w)
**Routine Clinical Activity**				
Clinical review to assess for suspected PM	X			
TUS assessment for LAT feasibility [a]	X			
Contrast-enhanced CT Thorax [b]	X			
Thoracoscopy (LAT /VATS) with pleural biopsies & fluid sent for diagnostic purposes [c]		X		
Chest Radiograph post-procedure (within 1-24h)		X		
Clinical review with results of pleural sampling			X	
**Study Activity**				
Review Eligibility Criteria	X			
If potentially eligible, introduce study [d], provide with PIS and discuss participation [e]	X			
Informed Written Consent	X	X [f]		
Register participant with CTU	X	X [f]		
Record Baseline Data [g]	X	X [f]		
Blood Sampling, Processing and Banking [h]	X	X [f]		
Exhaled Breath Sampling and Processing [i]	X	X [f]		
Acquisition and banking of multi-region pleural biopsies (4-6) and pleural fluid (100-500ml) for research analyses during LAT or VATS [c, h]		X		
Arrange retrieval of FFPE pleural biopsies from local pathology and transport to RTB [j]			X	
Record Follow-up Data		X	X	X [k, l]
Record Adverse Events		X	X	X

a. Not required if VATS thoracoscopy planned
b. A baseline contrast-enhanced CT Thorax should ideally be available within 12 weeks of visit B1 to confirm participant has not progressed since original benign biopsy. If not available, repeat CT should be considered based on clinical judgement but is not mandatory.
c. Please refer to Meso-ORIGINS Biopsy Manual
d. Investigators may introduce the study at earlier clinic visits if eligibility likely and clinically appropriate
e. Participants will be offered a follow-up telephone call with a member of the study team if they wish to have more time to consider the study. This call will occur no later than 2 working days after Visit provision of PIS.
f. If not already performed
g. Including the following baseline blood results from patient records, which should be repeated if not available within 4 weeks of visit B1: full blood count, lactate dehydrogenase, c-reactive protein, albumin, urea and electrolytes. Baseline data also includes results any previous pleural fluid and imaging tests performed as part of routine clinical care.
h. Please refer to Meso-ORIGINS Sample Handling Manual
i. Please refer to Meso-ORIGINS Exhaled Breath Sampling Manual. Exhaled breath samples can be omitted at sites where facilities are not in place for acquisition or storage, or on grounds of patient preference

j. FFPE biopsy blocks should be retrieved from the local pathology archive and transported to the PREDICT-Meso Research Tissue Bank (RTB), based in Glasgow. Please refer to the Meso-ORIGINS Sample Handling Manual for detailed instructions
k. If the patient is diagnosed with benign pleural inflammation and does NOT enter Arm A, a remote update regarding vital status and any new pleural diagnosis (e.g., mesothelioma) is required at 12-months
l. If the patient is diagnosed with mesothelioma, a remote update regarding treatments received, response and vital status is required at 12-months.
